# Confirmed Invasive Pulmonary Aspergillosis and COVID-19: the value of *postmortem* findings to support *antemortem* management

**DOI:** 10.1590/0037-8682-0401-2020

**Published:** 2020-07-03

**Authors:** Monique Freire Santana, Guilherme Pivoto, Márcia A. Araujo Alexandre, Djane Clarys Baía-da-Silva, Mayla Gabriela da Silva Borba, Fernando Almeida Val, Jose Diego Brito-Sousa, Gisely Cardoso Melo, Wuelton Marcelo Monteiro, João Vicente Braga Souza, Silviane Bezerra Pinheiro, Luiz Carlos Lima Ferreira, Felipe Gomes Naveca, Valdinete Alves Nascimento, André Lima Guerra Corado, Ludhmila Abrahão Hajjar, João Ricardo Silva, George Alan Villarouco Siva, Alessandro C. Pasqualotto, Marcus Vinícius Guimarães Lacerda

**Affiliations:** 1 Fundação de Medicina Tropical Dr. Heitor Vieira Dourado, Manaus, AM, Brasil.; 2 Universidade do Estado do Amazonas, Programa de Pós-Graduação em Medicina Tropical, Manaus, AM, Brasil.; 3 Departamento de Ensino e Pesquisa, Fundação Centro de Controle de Oncologia do Estado do Amazonas, Manaus, AM, Brasil.; 4 Hospital e Pronto Socorro Delphina Rinaldi Abdel Aziz, Manaus, AM, Brasil.; 5 Instituto Nacional de Pesquisas da Amazônia, Laboratório de Micologia, Manaus, AM, Brasil.; 6 Fundação Oswaldo Cruz, Instituto Leônidas & Maria Deane, Manaus, AM, Brasil.; 7 Fundação Oswaldo Cruz, Instituto Oswaldo Cruz, Programa de Pós-Graduação em Biologia Celular e Molecular, Rio de Janeiro, RJ, Brasil.; 8 Universidade de São Paulo, São Paulo, SP, Brasil.; 9 Universidade Federal de Ciências da Saúde de Porto Alegre, Porto Alegre, RS, Brasil.

**Keywords:** COVID-19, Aspergillus, Postmortem evidence

## Abstract

We present *postmortem* evidence of invasive pulmonary aspergillosis (IPA) in a patient with severe COVID-19. Autopsies of COVID-19 confirmed cases were performed. The patient died despite antimicrobials, mechanical ventilation, and vasopressor support. Histopathology and peripheral blood galactomannan antigen testing confirmed IPA. *Aspergillus penicillioides* infection was confirmed by nucleotide sequencing and BLAST analysis. Further reports are needed to assess the occurrence and frequency of IPA in SARS-CoV-2 infections, and how they interact clinically.

## INTRODUCTION

COVID-19 is characterized by asymptomatic and/or mild flu-like symptoms; however, about 20% of patients may progress to pneumonia and sepsis, thus requiring intensive support^1^. Patients with acute respiratory distress syndrome due to viral infections, such as SARS-CoV-2, are prone to secondary complications, including aspergillosis^2^. Invasive aspergillosis is not uncommon in immunocompromised patients, and is a well-described complication in acute respiratory failure and severe influenza-related pneumonia^3^. A retrospective multicenter cohort study showed that influenza infection was an independent risk factor for invasive pulmonary aspergillosis (IPA)^4^. 

Although influenza-associated IPA is associated with high mortality and morbidity, its pathogenesis is not well known^3^. Direct immunomodulation and the use of drugs, such as oseltamivir and corticosteroids, may play a role^5^ ^,^ ^6^. Wang et al.^2^ reported a patient with severe acute respiratory syndrome (SARS), who died of aspergillosis after prolonged treatment with corticosteroids. Blaize et al.^7^ and Antinori et al.^8^ reported fatal cases of IPA in immunocompetent patients with severe COVID-19, whose bronchoalveolar aspirate grew *Aspergillus fumigatus* and had a positive serum galactomannan (GM) antigen. Antinori et al.^8^ evidenced the pathological pulmonary alterations *postmortem* and confirmed the infective *Aspergillus* via molecular techniques. It is possible that aspergillosis could predispose patients with COVID-19 to clinical worsening.

Therefore, testing for the presence of *Aspergillus* spp in lower respiratory secretions and GM (not routinely positive in peripheral blood) in patients with COVID-19 in the intensive care unit (ICU) should be considered to allow timely treatment and avoid potential immunosuppression with the use of medications^5^ ^,^ ^6^. However, conducting a bronchoscopy in patients with COVID-19 is relatively contraindicated due to the biological risk and clinical deterioration caused by the procedure^9^. Lung biopsy, which might also be considered a gold standard diagnosis method, is also impractical in such a scenario.

Latin America seems to be the most recent COVID-19 epicenter, after Asia, Europe, and the US. In Manaus, in the Brazilian Amazon, more than 2,000 deaths were officially reported in April/May 2020.

## CASE REPORT

A 71-year-old male patient with prior history of hypertension, type II diabetes mellitus, and chronic kidney disease was admitted to the *Hospital e Pronto-Socorro Delphina Rinaldi Abdel Aziz*, a referral unit for the treatment of patients with COVID-19 in Manaus. The patient was transferred from another hospital, where he had already been diagnosed with COVID-19 by RT-qPCR. Upon admission into the ICU, he was placed under orotracheal intubation, received high-dose vasoactive drugs, was hemodynamically unstable, and presented cyanosis and cold extremities. He was administered high-flow norepinephrine (1.41 µg/kg/min) and placed on invasive mechanical ventilation under aspiration of an orotracheal tube with high parameters (positive end-expiratory pressure [PEEP] 8/FiO_2_ 60%/respiratory rate 26; volume 360). The PaO_2_/FiO_2_ ratio was 86.6. The patient received oseltamivir (75 mg twice daily) and chloroquine (450 mg twice on the first day) via a nasoenteral tube, IV azithromycin (500 mg/day), IV ceftriaxone (2g/day), IV furosemide (20mg TID), and prophylactic subcutaneous enoxaparin (40 mg/day). No corticosteroid drugs were used.

Laboratory parameters showed increased urea (360.7 mg/dL), creatinine (8.46 mg/dL), and C-reactive protein (12 mg/L). Normal values were seen for potassium and sodium (5.24 mmol/L; and 136.8 mmol/L, respectively). Leukocytes 6,530/µL, platelets 285x10^9^/µL, hemoglobin 11.2 g/dL, hematocrit 34.5%, neutrophilia (84%), and lymphopenia (12,8%) were also observed. Chest x-rays showed infiltrate and nodular consolidation in the right lower lobe. No CT scan was performed. Blood culture was negative for bacterial growth. 

On day three, following admission, the patient progressed with hemodynamic worsening and refractory shock, with irreversible hypotension and bradycardia. He died the following day. The autopsy was authorized by legal representatives (an informed consent form was signed), as the patient was enrolled prior to death in the *CloroCovid-19 Study* (ClinicalTrials.gov Identifier: NCT04323527, approved by the Brazilian National Ethics Review Board CAAE 30152620.1.0000.0005). An autopsy was performed in the same hospital by trained technicians and under strict biosafety rules.

**FIGURE 1:**
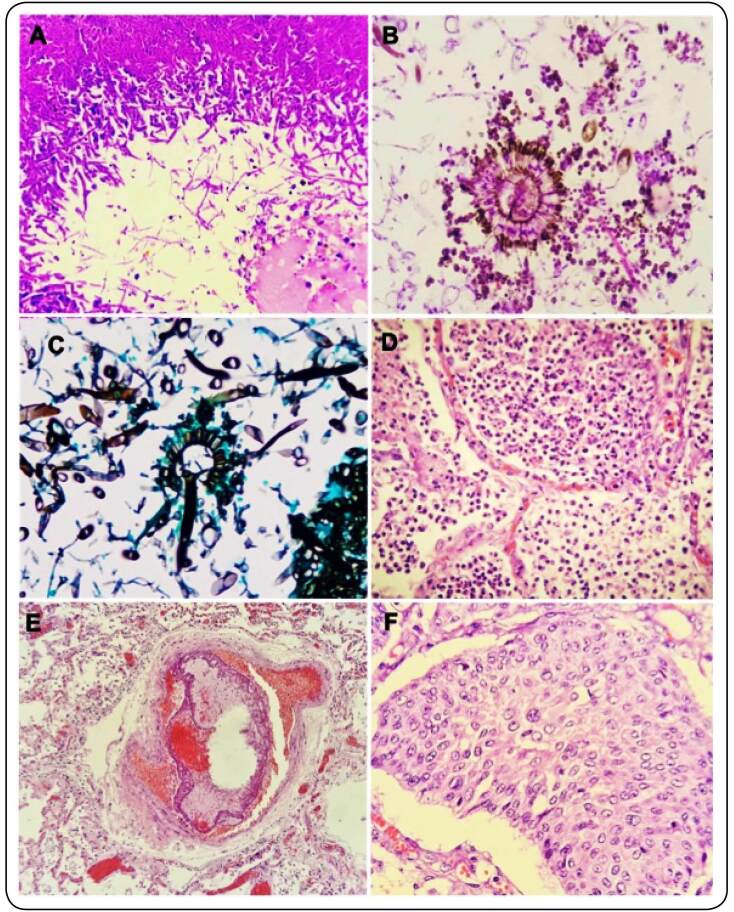
**Histopathology of the lung.** A. Numerous hyphae and fungal spores shown by H&E staining. Microscopic cavitation surrounded by numerous hypahes and fungal spores (40x). B. Well-defined Aspergillus head, allowing the visualization of phialides and conidia, with numerous fungal spores (PAS, 400x). C. Well-defined aspergillary structure showing conidiophore, aspergillary vesicle, phialides, and conidia, as well as several hyphae of regular diameter, some with septations and dichotomous branches (Gomori-Grocott, 400x). D. Bronchopneumonia with alveoli filled by neutrophils (H&E, 400x). E. Fibrin thrombi occluding a medium-sized artery (H&E, 400x) F. Squamous metaplasia in alveolar epithelial cells (H&E, 400x).

Macroscopically, the lung showed focal areas of consolidation in the right lower lobe. Microscopic visualization of the lung showed the presence of clearly defined *Aspergillus* structures, including hyphae and fungal spores, and a well-defined *Aspergillus* head with phialides, conidia, and spores, as well as bronchopneumonia, fibrin thrombi occluding an artery, and squamous metaplasia (Figure 1). The stored peripheral blood tested positive for the GM antigen (index 4.290). Since the diagnosis was made *postmortem*, and aspergillosis was not considered *antemortem*, no sputum was collected for fungus culture and no antifungal drugs were used.

The histopathological finding of *Aspergillus* spp was confirmed by nucleotide sequencing. The internal transcribed spacer 1 (ITS1) and ITS2 regions and the 5.8S ribosomal DNA (rDNA) were amplified by polymerase chain reaction using the universal primers ITS1 and ITS4, as described.^10^ Sequencing was performed using the BigDye^®^ Terminator v3.1 Cycle Sequencing (Applied Biosystems) and the above primers on an individual basis. Only the best sequences were used to perform contig assembly and conduct comparative analysis with the GenBank database using the Basic Local Alignment Search Tool. The best hit returns were *Aspergillus penicillioides*. The sequence was submitted to GenBank and received the accession number MT582426.

## DISCUSSION

Our report illustrates the importance of considering IPA in patients with underlying severe COVID-19, who show no clear clinical impairment related to IPA throughout their hospitalization. In this case, the patient was likely immunologically compromised due to his underlying conditions (Type II diabetes mellitus and chronic kidney disease). This case underscores the need to investigate this neglected disease and allow for early tracing with antifungal treatment; however, it is necessary to consider the risks posed by the bronchoscopy that must be performed for the diagnosis. Antinori et al.^8^ assessed the *postmortem* lung examination of a patient with IPA and COVID-19, in which IPA was characterized by bronchial wall ulceration associated with multiple spots of necrotizing pneumonia, and the residual lung parenchyma displayed a pattern of acute lung injury with diffuse alveolar damage. In addition to the presence of bronchopneumonia, we distinctly show fungi (hyphae and conidia) within blood vessels, the hallmark of the invasive disease triggered by this fungus. The presence of GM in bronchoalveolar lavage is a useful marker, but this procedure is not performed in many COVID-19 patients for biosafety reasons. However, the identification of this antigen in peripheral blood, a test that is usually negative, is seemingly a reliable marker of invasive disease, especially in our case, in which the invasion of vessels was observed.

In addition, it is important to consider that the use of medications, such as oseltamivir, which is preemptively prescribed for influenza infection, may potentially block host neuraminidase, thus increasing patient susceptibility to IPA^5^ ^,^ ^6^. The use of corticosteroids, which is still a matter of debate in COVID-19 treatment, may also facilitate invasive disease. Therefore, we recommend caution in the use of corticosteroids and oseltamivir to treat SARS-CoV-2 infection. Meanwhile, chloroquine has been proposed as a drug with antifungal and immune response properties^11^. As the patient clinical status was already critical upon his arrival and he died three days after hospitalization, the possibility of a hospital-acquired fungal infection is unlikely. It is possible that aspergillosis could predispose patients with COVID-19 to clinical worsening, and it is therefore necessary to further assess this two-way interaction between both infections.

*A. penicillioides* is a xerophilic species occurring in dry habitats and in house dust; it is responsible for human and animal allergies. Dry oxygen supplementation intended to prevent aerosolization could explain the infection caused by this pathogen^12^. To the best of our knowledge, this is the fourth clinical report on *A. penicillioides* clinical infection. The other three are classified as keratitis, disseminated disease in a child with cystic fibrosis, and central nervous system arteritis^13^ ^,^ ^14^ ^,^ ^15^. No information is available on the antifungal sensitivity of this species or the frequency of invasive disease.

In conclusion, we report a case of IPA in a patient with severe pneumonia associated with COVID-19. Using a gold standard method (histopathology) in our case series, the frequency of IPA was lower, as compared to that of cases published elsewhere; possible overestimation might be due to contamination. In our case, no CT imaging of the lung, sequential GM, or culture for fungus was performed, thus reinforcing the role of autopsies as a form of *postmortem* surveillance of such a severe disease. As the outbreak of COVID-19 continues to spread around the world, further reports are needed to assess the occurrence and frequency of IPA in severe SARS-CoV-2 infections, and their clinical interaction. These studies are needed to assess the incidence of IPA and define at-risk populations, thus offering a strategy for diagnosis, prophylaxis, and timely clinical management.
